# Liver transplantation for the treatment of iatrogenic bile duct injury

**DOI:** 10.1590/0100-6991e-20233565-en

**Published:** 2023-07-19

**Authors:** OLIVAL CIRILO, LUIZ EDUARDO RAFAEL MOUTINHO, PAULO SÉRGIO VIEIRA DE MELO, PRISCYLLA JENNIE MONTEIRO RABÊLO, AMERICO GUSMÃO AMORIM, CLÁUDIO MOURA LACERDA MELO

**Affiliations:** 1 - Universidade de Pernambuco, Faculdade de Ciências Médicas - Recife - PE - Brasil; 2 - Hospital Universitário Oswaldo Cruz, Unidade de Transplante de Fígado - Recife - PE - Brasil

## COMMENTARY

We read with interest the article entitled “Liver transplantation in the treatment of iatrogenic bile duct injury”, published in 2022 by Zeni et al.^1^, and we congratulate the team of authors and the Liver Transplant Service of the Federal University of Paraná for their work. The topic is of great relevance for the Brazilian surgical community. Liver transplantation (LT) is an established practice in Brazil and of utmost importance for end-stage liver disease. The work notably highlights the role of this therapeutic modality in iatrogenic bile duct injury (IBDI), especially after cholecystectomy.

As a contribution to the theme, it is worth highlighting, in contrast to the survey by Zeni et al.^1^, that currently the Brazilian literature comprises three publications on the subject, with 30 patients submitted to LT secondary to IBDI^1^ ^-^ ^3^. Our experience was published in 2017 in the journal GED - Gastroenterologia Endoscopia Digestiva -, under the title: “Liver transplantation in the treatment of iatrogenic bile duct injury after cholecystectomy: a study in a reference center in Northeast Brazil”^2^. In 2019, the center of the Federal University of Ceará published another retrospective case series^3^. Considering that LT is performed in a population restricted to a small sample and encompasses an extreme therapeutic measure to IBDI, transplant services in Brazil should be encouraged to communicate their experiences.

The United States (n=61)^4^, France (n=30)^5^, Argentina (n=19)^6^, Spain (n=24)^7^, and Europe/plurinational (n=34)^8^ have the highest multicentric series on this topic. The French study pointed to an increase in the incidence of LT for IBDI, in contrast to other parts of the world, which reported a decrease throughout the 1990s until the 2000s^6^ ^,^ ^7^. The diffusion of new complementary techniques and the improvement of follow-up of complex IBDI may have contributed to this fact and demonstrates a tendency towards a decrease in end-stage liver failure secondary to this type of injury.

There is a prevalence of open cholecystectomy in cases published in Brazil due to the absolute incidence of this approach in our country. The open technique predominates in this group of patients, since the surgery that caused the injury was usually performed in less complex centers and with less availability of minimally invasive surgery. All transplanted patients in the work by Zeni et al.^1^ and in ours^2^ were referred from other centers.

The complexity of managing IBDI, associated with the lack of resolution and the delay in referral to a center specialized in advanced hepatobiliary surgery, are important factors in the evolution of the disease’s natural history^5^ ^,^ ^8^ ^,^ ^9^. In fact, as cited by Silva Filho et al.^3^, biliary obstruction without adequate treatment can result in periportal fibrosis, hepatic fibrosis, and, subsequently, cirrhosis in respectively four, 22, and 62 months^10^. The time reported in Brazil for performing LT after the initial injury was on average 165, 140, and 62.2 months, respectively, in the studies by Fonseca Neto et al.^2^, de Silva Filho et al.^3^, and Zeni et al.^1^. These data coincide with the experience of international centers.

Several techniques can be employed to treat IBDI. Biliodigestive bypass with hepaticojejunostomy is the most used surgical approach, with the purpose of definitively reestablishing bile flow. However, endoscopic and radiological treatments stand out with success in managing this complication. The most severe and refractory cases may require multiple approaches, with different therapeutic modalities. Silva Filho et al.^3^ reported an average of 3.5 surgeries and procedures before LT. In our study, we pointed out that, prior to LT, two patients had not undergone any surgical procedures, five underwent biliodigestive bypasses, and one was approached with choledochal suture and papillotomy. In the series by Zeni et al.^1^, all patients had undergone at least one surgical procedure. However, there is no robust association between the prognosis of these patients and the number of previous biliary repairs, the level of injury, the time of injury, or the initial repair method^11^. The concomitant vascular injury exceptionally reduced the time to LT^5^ ^,^ ^8^. The extent of the initial injury and the involvement of the junction of the right and left hepatic ducts can be considered, though the indication of LT is reserved for patients with the development of end-stage liver disease or acute liver failure due to this type of injury.

Together, the three studies surveyed indicated that there were 11 deaths, with an overall mortality of 36%. Among the causes, early hemorrhagic shock prevailed (five patients). An analysis that included eight large LT European centers found an overall mortality of 29.4%^8^. A study from the University of Kentuchy assessed the United Network for Organ Sharing (UNOS) database and observed that IBDI was a predictor of early graft function failure and mortality in LT^4^. Multiple previous interventions are a differential factor in this group of patients and may partially explain such results.

Therefore, I conclude that IBDI is a potentially serious abdominal surgical complication that can culminate in recurrent cholangitis, liver fibrosis, and secondary biliary cirrhosis. Brazilian transplant centers have experience in managing this condition. We thank the authors for their timely contribution to the topic and we consider new national publications on IBDI and the role of LT in these patients as necessary.
